# Development and internal validation of a nomogram for early prediction of deep vein thrombosis after liver transplantation: an exploratory machine-learning comparison

**DOI:** 10.3389/fmed.2026.1863619

**Published:** 2026-07-29

**Authors:** Xintao Chen, Wen Luo, Lingxiang Xu, Mingxiang Cheng

**Affiliations:** Department of Hepatobiliary Surgery, The Second Affiliated Hospital of Chongqing Medical University, Chongqing, China

**Keywords:** deep vein thrombosis, liver transplantation, machine learning, nomogram, risk prediction

## Abstract

**Background:**

Lower-extremity deep vein thrombosis (DVT) after liver transplantation (LT) may delay recovery and complicate thromboprophylaxis. Practical transplant-specific tools for early risk stratification remain limited.

**Methods:**

Adult recipients who underwent LT between August 2023 and January 2026 were retrospectively screened. After exclusions, 131 recipients were divided by outcome-stratified random sampling into a training cohort (*n* = 99) and a hold-out subset (*n* = 32). The outcome was newly detected lower-extremity DVT identified by bilateral duplex color Doppler ultrasonography within the first 7 postoperative days. Postoperative laboratory variables were obtained from the first urgent blood test, generally within 1 h after completion of LT and before initiation of postoperative pharmacologic anticoagulation according to routine workflow. Least absolute shrinkage and selection operator regression was used for penalized feature selection, followed by multivariable logistic regression and nomogram construction. Random forest, support vector machine, LightGBM, and XGBoost were evaluated as exploratory comparators. Performance was assessed using discrimination, threshold-dependent classification measures, calibration, the Hosmer-Lemeshow test, decision curve analysis, stratified 5-fold cross-validation, and bootstrap optimism correction.

**Results:**

DVT occurred in 26 of 131 recipients (19.8%). The logistic model included postoperative ln (D-dimer), anhepatic phase, postoperative activated partial thromboplastin time, and preoperative prothrombin time. Its AUC was 0.79 (95% CI, 0.67–0.90) in the training cohort and 0.81 (95% CI, 0.56–1.00) in the hold-out subset. The 5-fold cross-validated AUC was 0.76 (95% CI, 0.63–0.86), and the bootstrap optimism-corrected AUC was 0.76. Wide, overlapping confidence intervals and model-specific optimism did not support reliable algorithm ranking.

**Conclusion:**

A concise logistic regression-based nomogram using routinely available perioperative variables may support early individualized DVT risk estimation after LT. The machine-learning analyses should be regarded as exploratory. Larger prospective multicenter studies are required before clinical implementation.

## 1 Introduction

Lower extremity deep vein thrombosis (DVT) after liver transplantation (LT) is an important postoperative complication because it may prolong hospitalization, complicate decisions regarding thromboprophylaxis, and increase the risk of pulmonary embolism and other adverse recovery events (1, 2). Patients with advanced liver disease are no longer regarded as simply “auto-anticoagulated”; rather, growing evidence supports a fragile state of rebalanced hemostasis in which bleeding and thrombosis may coexist (3, 4). In this context, thrombotic risk after LT deserves specific attention and should not be underestimated solely on the basis of conventional coagulation abnormalities (4, 5).

The peri-transplant period exposes recipients to several prothrombotic and counter-regulatory influences. Ischemia–reperfusion injury, endothelial activation, central venous catheterization, prolonged immobilization, major blood product exposure, and marked hemodynamic fluctuation may all contribute to venous thrombosis after LT (6). Previous studies have shown that DVT and pulmonary thromboembolism are not rare after LT, although reported incidences vary across cohorts because of differences in screening intensity, prophylaxis strategies, and outcome definitions (7–9). Risk assessment in LT recipients therefore cannot simply mirror that used for general surgical populations, because transplant surgery combines distinctive disturbances in coagulation, portal hemodynamics, graft ischemia, and early postoperative organ dysfunction (10–12).

At the same time, thromboprophylaxis after LT remains controversial. Some recipients may benefit from closer surveillance and preventive intervention, whereas routine aggressive anticoagulation is difficult to justify because postoperative bleeding, graft vascular complications, and marked inter-patient heterogeneity in coagulation status must also be considered (12, 13). Recent expert statements emphasize individualized rather than uniform management (13, 14). Venous thromboembolism has also been documented across solid-organ transplant populations (15). A concise model based on routinely available perioperative variables could therefore help identify patients who may require intensified Doppler surveillance, earlier mobilization, or multidisciplinary reassessment of prophylaxis. In the present study, we developed and internally validated an early prediction model for lower extremity DVT after LT, translated the final multivariable logistic model into a nomogram, and explored its performance alongside several machine-learning algorithms without presuming that any single algorithm was superior.

## 2 Materials and methods

### 2.1 Study design and patient selection

This retrospective cohort study included adult recipients who underwent LT at The Second Affiliated Hospital of Chongqing Medical University between August 2023 and January 2026. A total of 145 recipients were screened. Patients were excluded because of pre-existing lower-extremity thrombosis (*n* = 1), missing preoperative ultrasound data (*n* = 4), or missing postoperative ultrasound data (*n* = 9). The final analytical cohort comprised 131 recipients. Using outcome-stratified random sampling with a fixed seed of 2025, recipients were divided at a ratio of 3:1 into a training cohort and a hold-out subset. Study design, analysis, and reporting were developed with reference to the Transparent Reporting of a Multivariable Prediction Model for Individual Prognosis or Diagnosis recommendations (16, 17). The study flow is shown in Supplementary Figure S1.

The study protocol conformed to the ethical guidelines of the 1975 Declaration of Helsinki and was approved by the Ethics Committee of The Second Affiliated Hospital of Chongqing Medical University [Pre-review No. (2025)361]. Given the retrospective observational design and anonymized analysis, the requirement for individual informed consent was waived according to institutional policy.

### 2.2 Liver transplantation

Organs were obtained from deceased donors following brain-death determination and were allocated through the China Organ Transplant Response System in accordance with applicable Chinese regulations; no organs from executed prisoners were used. Liver transplantations were performed by an experienced surgical team using classic or piggyback techniques.

### 2.3 Outcome definition and variable collection

The primary outcome was newly detected lower-extremity DVT during the first 7 days after LT. Preoperative bilateral lower-extremity duplex ultrasonography was performed to exclude pre-existing thrombosis. Candidate variables included demographic characteristics, comorbidities, liver disease severity indices, preoperative laboratory measurements, inflammatory and nutritional indices, intraoperative variables, and postoperative laboratory variables. All postoperative laboratory variables used in the analysis were obtained from the first urgent blood test after completion of LT, generally within 1 h, thereby positioning the model as an immediate postoperative risk-assessment tool.

### 2.4 Ultrasound surveillance and DVT diagnosis

All recipients included in the analytical cohort underwent preoperative bilateral lower-extremity duplex ultrasonography and at least one postoperative bilateral examination within the first 7 days after LT. Postoperative examinations were not performed on a fixed daily schedule; their timing and any repeat examinations were determined by routine postoperative assessment and the patient’s clinical condition. According to routine departmental practice, trained sonographers used compression, color-flow, and spectral Doppler techniques to assess bilateral proximal and distal deep venous segments. DVT was diagnosed on the basis of incomplete or absent venous compressibility or direct visualization of an intraluminal thrombus; abnormal or absent Doppler flow was used as supportive evidence. A thrombus detected postoperatively that was absent on the preoperative examination was classified as postoperative DVT. Because examination timing and frequency were not uniform, potential variation in event ascertainment was considered in the interpretation of the results.

### 2.5 Perioperative thromboprophylaxis and anticoagulation

Postoperative thromboprophylaxis and anticoagulation were not governed by a uniform regimen and were individualized by the transplant team according to the balance between bleeding and thrombotic risk. Decisions generally considered surgical hemostasis and drain output, platelet count, renal and graft function, vascular reconstruction, Doppler findings, and pre-existing thrombotic indications; pre-existing portal vein thrombosis and complex portal reconstruction could also influence antithrombotic management (12–14). Therapeutic anticoagulation was used for confirmed thrombosis or selected vascular indications after multidisciplinary assessment. Because prophylaxis exposure was not recorded in a sufficiently standardized form for retrospective modeling, it was not included as a predictor. According to routine workflow, the first urgent postoperative blood sample used in the present analysis was generally collected within 1 h after LT and before initiation of postoperative pharmacologic anticoagulation. However, exact exposure to intraoperative or very early postoperative anticoagulants could not be reconstructed for every recipient. This timing reduces concern that postoperative APTT was primarily determined by postoperative anticoagulant exposure but does not exclude effects of intraoperative anticoagulants, transfusion, factor consumption, or other perioperative processes.

### 2.6 Model development and evaluation

In the training cohort, candidate variables were first compared between recipients with and without DVT using the statistical tests described below. Variables with a nominal *p* < 0.05 in these exploratory comparisons were entered into least absolute shrinkage and selection operator (LASSO) regression. The LASSO model used a binomial likelihood, an L1 penalty (alpha = 1), standardized predictors, and 10-fold cross-validation with binomial deviance as the selection criterion (18). The tuning parameter was set at lambda.min, corresponding to the minimum mean cross-validated deviance. Five predictors had nonzero coefficients at lambda.min: postoperative ln (D-dimer), anhepatic phase, intraoperative plasma transfusion, postoperative APTT, and preoperative PT. These five predictors were then evaluated using univariable logistic regression, and variables with *p* < 0.10 were entered into the multivariable logistic regression model used to construct the nomogram. Continuous predictors were retained on their continuous scales; no data-driven dichotomization or predictor-specific cut-off selection was performed. D-dimer, AST, and ALT were natural-log transformed because of skewed distributions. This staged, data-driven selection process was considered exploratory and its potential for selection-related optimism was addressed in the limitations.

The final four-predictor set was used consistently for logistic regression and the four exploratory machine-learning models. Logistic regression used a binomial logit link. Random forest used 500 trees, mtry = 1, class-balanced bootstrap sampling with the per-class sample size set to 80% of the minority-class count, a terminal-node size of 8, and a maximum of 6 terminal nodes; out-of-bag votes were used for its training-cohort probability estimates. The support vector machine used a radial-basis-function kernel with predictor scaling, cost = 0.5, and gamma = 0.10. LightGBM used a learning rate of 0.02, 7 leaves, maximum depth 2, minimum leaf size 15, feature fraction 0.65, bagging fraction 0.70 with bagging at every iteration, minimum split gain 0.10, L1 regularization 0.8, L2 regularization 3, and 120 boosting rounds. XGBoost used eta = 0.02, maximum depth 2, minimum child weight 5, subsample 0.70, column subsampling 0.65, gamma 0.5, L2 regularization 3, L1 regularization 1, maximum delta step 1, and 120 boosting rounds. No data-driven hyperparameter search was performed; the settings were fixed in the final analysis to constrain model complexity in the small-event dataset.

Discrimination was assessed using receiver operating characteristic (ROC) analysis and the area under the ROC curve (AUC). Training-cohort and hold-out AUC confidence intervals were obtained using the DeLong method. The empirical ROC objects were used for AUC estimation, whereas binormal smoothing was applied only to the displayed ROC curves. Model-specific probability thresholds were selected using the Youden index solely to calculate accuracy, sensitivity, specificity, positive predictive value, and negative predictive value; these thresholds were not used to dichotomize continuous predictors. Calibration was assessed using grouped calibration plots, Brier score, logistic recalibration intercept and slope, and the Hosmer-Lemeshow goodness-of-fit test. Depending on sample size, predicted probabilities were divided into four to six equal-frequency groups; observed proportions were displayed with approximate normal 95% intervals, and monotone interpolation was used only to aid visualization. The Hosmer-Lemeshow analysis used 10 equal-frequency groups, with automatic reduction when required by sample size. Decision curve analysis (DCA) estimated net benefit across threshold probabilities from 0.01 to 0.70 (19, 20); model curves were smoothed using locally estimated scatterplot smoothing only for graphical display. Because the hold-out subset contained only six DVT events, threshold-dependent classification and numerical calibration measures were reported only for the training cohort and as descriptive pooled estimates for the full analytical cohort. The pooled estimates were generated by applying the training-fitted models to all 131 recipients; because they combine development and hold-out observations, they were not interpreted as independent validation. Separate Youden thresholds were estimated in the training cohort and the pooled analytical cohort. Additional internal validation used the fixed four-predictor set in stratified 5-fold cross-validation across the entire cohort, with models refitted in each fold and performance calculated from out-of-fold probabilities. Cross-validated AUC 95% confidence intervals were estimated by 2,000 stratified bootstrap replicates of the paired outcome and out-of-fold predictions. Bootstrap optimism correction used 200 resamples: each model was fitted in a bootstrap sample, evaluated in that sample and in the original dataset, and the mean AUC difference was subtracted from the full-data apparent AUC. Predictor selection was not repeated within the cross-validation or bootstrap loops. For resampling, random forest used 250 trees with fold-specific node size, LightGBM and XGBoost used 80 boosting rounds, LightGBM minimum leaf size was adapted to fold size, and XGBoost used fold-specific class weighting; the remaining stated settings were retained. Shapley additive explanations (SHAP) were calculated from the originally developed training-cohort LightGBM model using contribution values on the raw-score scale and were aggregated from encoded features to their parent variables. SHAP was used only for exploratory *post hoc* interpretation and not for predictor selection or nomogram construction (21).

### 2.7 Statistical analysis

Continuous variables are presented as mean ± standard deviation or median (Q1, Q3), as appropriate, and categorical variables as number (percentage). Distributional form was assessed separately within each cohort; consequently, a variable may be summarized as mean ± standard deviation in one cohort and as median (Q1, Q3) in the other. Between-group comparisons were performed using Welch’s t test, the Wilcoxon rank-sum test, the chi-square test, or Fisher’s exact test, as appropriate. Because only six DVT events occurred in the hold-out subset, DVT-versus-non-DVT *p* values were reported only for the training cohort; hold-out data were summarized descriptively. Patients lacking the required preoperative or postoperative ultrasound assessment were excluded before modeling. Completeness of all candidate predictors was checked after these exclusions; no candidate predictor values were missing in the final analytical dataset, and no imputation was performed. Potential collinearity was assessed before LASSO using pairwise Spearman correlation coefficients and variance inflation factors (VIFs), with |r| ≥ 0.70 or VIF ≥ 5 considered potentially important. LASSO regularization provided an additional safeguard against redundant predictors. All tests were two-sided, and *p* < 0.05 was considered statistically significant except for the prespecified *p* < 0.10 criterion used to advance LASSO-selected variables from univariable to multivariable logistic regression. Analyses were performed using R version 4.5.2 (R Foundation for Statistical Computing, Vienna, Austria).

## 3 Results

### 3.1 Cohort characteristics

A total of 131 adult LT recipients were included in the final analysis, including 99 in the training cohort and 32 in the hold-out subset. DVT was detected within the first 7 postoperative days in 20 recipients in the training cohort and 6 recipients in the hold-out subset, corresponding to an overall detected DVT rate of 19.8%. Baseline, preoperative, intraoperative, and immediate postoperative characteristics stratified by DVT status are presented in Tables 1–3.

**Table 1:** Baseline and clinical characteristics.

Characteristic	Level	Training overall (*n* = 99)	Training non-DVT (*n* = 79)	Training DVT (*n* = 20)	*p* value	Hold-out overall (*n* = 32)	Hold-out non-DVT (*n* = 26)	Hold-out DVT (*n* = 6)
Demographic and clinical characteristics								
Sex	Male	75 (75.8%)	61 (77.2%)	14 (70.0%)	0.562	25 (78.1%)	21 (80.8%)	4 (66.7%)
	Female	24 (24.2%)	18 (22.8%)	6 (30.0%)		7 (21.9%)	5 (19.2%)	2 (33.3%)
Smoking history	No	62 (62.6%)	49 (62.0%)	13 (65.0%)	0.806	20 (62.5%)	16 (61.5%)	4 (66.7%)
	Yes	37 (37.4%)	30 (38.0%)	7 (35.0%)		12 (37.5%)	10 (38.5%)	2 (33.3%)
Height (cm)		168.00 (160.00, 170.00)	168.00 (160.00, 170.50)	166.00 (159.75, 170.00)	0.468	170.00 (161.75, 172.00)	169.00 (162.75, 170.75)	171.50 (159.75, 172.75)
Weight (kg)		63.68 ± 12.56	63.23 ± 12.56	65.45 ± 12.72	0.490	63.22 ± 10.71	64.04 ± 11.13	59.67 ± 8.55
Diabetes	No	85 (85.9%)	68 (86.1%)	17 (85.0%)	1.000	26 (81.2%)	21 (80.8%)	5 (83.3%)
	Yes	14 (14.1%)	11 (13.9%)	3 (15.0%)		6 (18.8%)	5 (19.2%)	1 (16.7%)
Prior surgery	No	34 (34.3%)	26 (32.9%)	8 (40.0%)	0.551	11 (34.4%)	9 (34.6%)	2 (33.3%)
	Yes	65 (65.7%)	53 (67.1%)	12 (60.0%)		21 (65.6%)	17 (65.4%)	4 (66.7%)
Hypertension	No	87 (87.9%)	71 (89.9%)	16 (80.0%)	0.255	26 (81.2%)	20 (76.9%)	6 (100.0%)
	Yes	12 (12.1%)	8 (10.1%)	4 (20.0%)		6 (18.8%)	6 (23.1%)	0 (0.0%)
Coronary heart disease	No	96 (97.0%)	76 (96.2%)	20 (100.0%)	1.000	32 (100.0%)	26 (100.0%)	6 (100.0%)
	Yes	3 (3.0%)	3 (3.8%)	0 (0.0%)				
MELD score		14.00 (9.00, 24.50)	13.00 (9.00, 22.50)	18.50 (11.50, 30.25)	**0.029**	18.19 ± 8.28	17.92 ± 8.29	19.33 ± 8.91
Age category	≤60 years	89 (89.9%)	71 (89.9%)	18 (90.0%)	1.000	28 (87.5%)	23 (88.5%)	5 (83.3%)
	>60 years	10 (10.1%)	8 (10.1%)	2 (10.0%)		4 (12.5%)	3 (11.5%)	1 (16.7%)
BMI category	≤24 kg/m^2^	60 (60.6%)	51 (64.6%)	9 (45.0%)	0.110	21 (65.6%)	15 (57.7%)	6 (100.0%)
	>24 kg/m^2^	39 (39.4%)	28 (35.4%)	11 (55.0%)		11 (34.4%)	11 (42.3%)	0 (0.0%)
Child–Pugh grade	A	23 (23.2%)	21 (26.6%)	2 (10.0%)	0.192	4 (12.5%)	4 (15.4%)	0 (0.0%)
	B	32 (32.3%)	26 (32.9%)	6 (30.0%)		15 (46.9%)	12 (46.2%)	3 (50.0%)
	C	44 (44.4%)	32 (40.5%)	12 (60.0%)		13 (40.6%)	10 (38.5%)	3 (50.0%)
Surgical technique	Classic	79 (79.8%)	66 (83.5%)	13 (65.0%)	0.114	28 (87.5%)	23 (88.5%)	5 (83.3%)
	Piggyback	20 (20.2%)	13 (16.5%)	7 (35.0%)		4 (12.5%)	3 (11.5%)	1 (16.7%)

**Table 2:** Preoperative and intraoperative characteristics.

Characteristic	Level	Training overall (*n* = 99)	Training non-DVT (*n* = 79)	Training DVT (*n* = 20)	*P*-value	Hold-out overall (*n* = 32)	Hold-out non-DVT (*n* = 26)	Hold-out DVT (*n* = 6)
Preoperative laboratory variables								
Preoperative neutrophil count (×10^9/L)	2.70 (1.69, 4.47)	2.69 (1.83, 4.00)	4.32 (1.37, 6.33)	0.486	2.85 (1.76, 5.25)	3.29 (1.90, 5.08)	2.38 (1.69, 6.75)	
Preoperative lymphocyte count (×10^9/L)	0.59 (0.44, 1.11)	0.58 (0.44, 0.88)	0.92 (0.30, 1.37)	0.464	0.68 (0.45, 0.88)	0.65 (0.46, 0.84)	0.90 (0.39, 1.14)	
Preoperative monocyte count (×10^9/L)	0.33 (0.24, 0.51)	0.32 (0.24, 0.46)	0.48 (0.23, 0.93)	0.137	0.41 (0.24, 0.55)	0.35 (0.25, 0.50)	0.55 (0.21, 0.80)	
Preoperative platelet count (×10^9/L)	67.00 (47.00, 104.50)	67.00 (42.00, 97.50)	69.00 (50.00, 137.50)	0.469	61.50 (35.75, 99.00)	61.50 (41.25, 119.25)	53.50 (34.50, 72.50)	
Preoperative CRP (mg/L)	9.03 (5.00, 20.97)	8.09 (5.00, 20.63)	12.90 (7.60, 20.91)	0.239	8.12 (5.00, 26.25)	9.10 (5.00, 27.32)	5.21 (5.00, 15.98)	
Preoperative hemoglobin (g/L)	103.62 ± 24.35	104.23 ± 25.01	101.20 ± 21.97	0.596	105.50 ± 21.26	107.65 ± 21.05	96.17 ± 21.40	
Preoperative albumin (g/L)	35.24 ± 6.12	35.56 ± 6.30	33.98 ± 5.35	0.262	34.42 ± 4.99	34.94 ± 4.84	32.13 ± 5.47	
Preoperative eGFR (mL/min/1.73 m^2^)	107.20 (90.45, 118.10)	106.70 (94.10, 118.80)	110.00 (86.95, 116.18)	0.951	105.15 (88.78, 117.65)	101.80 (87.32, 117.22)	112.00 (98.30, 117.00)	
Preoperative creatinine (μmol/L)	63.85 (52.47, 76.38)	63.85 (52.47, 74.40)	64.25 (52.35, 85.40)	0.923	64.45 (50.32, 76.75)	68.70 (50.83, 79.75)	54.55 (44.07, 64.58)	
Preoperative red blood cell count (×10^12/L)	3.43 (2.68, 4.08)	3.43 (2.72, 4.08)	3.26 (2.48, 3.97)	0.433	3.47 ± 0.62	3.49 ± 0.65	3.40 ± 0.53	
Preoperative total bilirubin (μmol/L)	44.70 (20.40, 178.15)	37.20 (18.70, 155.30)	88.15 (27.75, 335.88)	0.115	51.90 (30.08, 308.27)	50.90 (30.13, 275.17)	74.65 (25.90, 267.62)	
Preoperative PT (s)	16.60 (14.45, 21.70)	16.30 (14.30, 20.70)	18.85 (15.60, 25.48)	**0.046**	17.00 (15.05, 22.45)	16.40 (14.45, 20.30)	20.75 (18.50, 25.10)	
Preoperative inflammatory/Nutritional indices								
Preoperative SII	320.19 (166.18, 639.47)	299.53 (161.63, 619.39)	496.33 (194.31, 830.97)	0.306	339.90 (161.32, 573.80)	339.90 (186.65, 669.54)	302.75 (172.99, 462.32)	
Preoperative CLR	16.81 (7.86, 42.18)	16.72 (7.39, 37.42)	17.27 (9.92, 55.99)	0.568	17.35 (9.95, 42.15)	17.35 (10.14, 44.45)	19.03 (8.39, 26.48)	
Preoperative NLR	3.92 (2.43, 6.53)	3.87 (2.43, 6.53)	4.37 (2.64, 7.33)	0.660	6.32 ± 3.70	6.36 ± 3.77	6.18 ± 3.71	
Preoperative PLR	102.78 (71.12, 183.71)	96.30 (73.71, 173.01)	115.39 (47.94, 252.54)	0.744	92.75 (71.26, 143.48)	109.41 (82.54, 155.07)	72.29 (58.24, 124.48)	
Preoperative SIRI	1.24 (0.59, 3.23)	1.16 (0.64, 2.60)	1.98 (0.45, 8.16)	0.362	1.83 (0.82, 4.32)	1.89 (0.68, 4.44)	1.71 (0.99, 3.63)	
Preoperative PNI	38.85 (34.02, 43.17)	39.00 (33.92, 43.33)	37.77 (34.65, 41.74)	0.682	36.47 (33.41, 41.45)	37.50 (34.61, 42.35)	33.12 (31.69, 37.90)	
Preoperative CAR	0.30 (0.16, 0.57)	0.25 (0.14, 0.57)	0.40 (0.26, 0.57)	0.142	0.25 (0.15, 0.81)	0.27 (0.14, 0.85)	0.17 (0.16, 0.49)	
Preoperative SIRI-P	92.77 (44.57, 315.64)	86.55 (48.35, 252.93)	307.20 (24.98, 598.16)	0.135	128.12 (28.76, 352.83)	128.12 (27.95, 358.22)	93.85 (35.03, 263.90)	
Intraoperative variables								
Operation time (min)	435.00 (400.00, 507.50)	430.00 (390.50, 496.50)	452.50 (411.25, 520.00)	0.170	442.62 ± 84.48	447.08 ± 83.51	423.33 ± 93.97	
Anhepatic phase (min)	66.00 (55.00, 79.50)	65.00 (53.00, 78.00)	78.00 (61.00, 83.75)	**0.024**	62.75 ± 17.73	61.04 ± 15.95	70.17 ± 24.41	
Intraoperative fluid (mL)	3800.00 (3300.00, 4475.00)	3800.00 (3175.00, 4425.00)	3762.50 (3350.00, 4781.25)	0.353	3966.80 ± 912.36	4061.06 ± 840.00	3558.33 ± 1177.46	
Intraoperative urine output (mL)	2196.21 ± 1104.70	2126.46 ± 1046.06	2471.75 ± 1304.65	0.283	1927.97 ± 1059.41	2017.12 ± 1049.17	1541.67 ± 1111.04	
Blood loss (mL)	800.00 (600.00, 1000.00)	800.00 (600.00, 1000.00)	1000.00 (800.00, 1275.00)	0.066	800.00 (750.00, 1000.00)	800.00 (800.00, 1000.00)	1000.00 (700.00, 1000.00)	
Intraoperative plasma transfusion (U)	1.50 (0.00, 2.00)	0.00 (0.00, 2.00)	2.00 (0.00, 3.00)	**0.040**	1.50 (0.00, 2.00)	1.00 (0.00, 2.00)	1.50 (0.25, 3.50)	
Intraoperative red blood cell transfusion (U)	3.50 (0.00, 6.00)	3.00 (0.00, 5.00)	5.25 (0.00, 8.00)	0.067	3.00 (2.00, 4.00)	3.00 (1.62, 4.00)	2.75 (2.12, 3.75)	

**Table 3:** Immediate postoperative characteristics.

Characteristic	Level	Training overall (*n* = 99)	Training non-DVT (*n* = 79)	Training DVT (*n* = 20)	*P* value	Hold-out overall (*n* = 32)	Hold-out non-DVT (*n* = 26)	Hold-out DVT (*n* = 6)
Postoperative laboratory variables								
Postoperative APTT (s)		64.40 (52.20, 79.20)	63.80 (51.30, 76.10)	71.25 (59.55, 156.52)	**0.041**	64.10 (50.20, 75.75)	58.70 (49.00, 72.58)	109.00 (67.77, 171.82)
Postoperative PTA (%)		38.23 ± 12.29	38.65 ± 12.64	36.60 ± 10.91	0.474	36.78 ± 11.51	37.08 ± 10.52	35.50 ± 16.32
Postoperative neutrophil count (×10^9/L)		8.33 (6.11, 10.72)	8.40 (5.91, 10.22)	7.96 (6.46, 15.14)	0.505	7.84 (5.16, 11.21)	7.75 (4.82, 9.40)	13.56 (8.59, 14.42)
Postoperative lymphocyte count (×10^9/L)		0.33 (0.24, 0.47)	0.33 (0.23, 0.46)	0.34 (0.29, 0.72)	0.226	0.24 (0.17, 0.39)	0.24 (0.19, 0.38)	0.26 (0.14, 0.42)
Postoperative monocyte count (×10^9/L)		0.39 (0.24, 0.60)	0.38 (0.25, 0.58)	0.50 (0.24, 0.79)	0.245	0.34 (0.22, 0.62)	0.32 (0.23, 0.61)	0.44 (0.20, 0.63)
Postoperative platelet count (×10^9/L)		54.00 (35.00, 80.00)	54.00 (33.00, 81.00)	49.00 (38.00, 70.25)	0.744	51.00 (32.00, 63.50)	51.50 (37.50, 64.50)	36.00 (26.25, 50.25)
Postoperative CRP (mg/L)		49.73 (22.55, 86.69)	49.73 (25.16, 83.31)	46.31 (10.81, 88.57)	0.556	52.22 (21.99, 82.39)	52.22 (26.14, 79.02)	50.02 (15.89, 112.83)
Postoperative hemoglobin (g/L)		83.05 ± 15.39	83.67 ± 16.18	80.60 ± 11.76	0.343	79.62 ± 14.74	78.65 ± 14.27	83.83 ± 17.41
Postoperative albumin (g/L)		39.60 (35.05, 42.60)	40.10 (35.85, 43.05)	38.65 (32.85, 41.10)	0.209	37.71 ± 4.51	38.37 ± 4.27	34.88 ± 4.77
Postoperative eGFR (mL/min/1.73 m^2^)		95.10 (62.25, 112.45)	93.10 (62.25, 110.55)	96.85 (75.25, 119.88)	0.539	102.60 (69.73, 116.08)	103.35 (74.78, 116.22)	73.40 (69.38, 100.60)
Postoperative total bilirubin (μmol/L)		75.20 (41.40, 135.75)	71.80 (40.10, 126.20)	111.90 (61.02, 220.05)	0.054	74.45 (53.78, 138.83)	69.90 (52.92, 129.48)	104.80 (73.40, 202.35)
Postoperative ln(D-dimer)		7.72 (6.96, 8.62)	7.55 (6.71, 8.32)	8.38 (7.66, 9.20)	**0.004**	7.34 ± 1.26	7.13 ± 1.20	8.21 ± 1.19
Postoperative ln(AST)		7.28 ± 0.85	7.23 ± 0.82	7.48 ± 0.97	0.296	7.20 (6.76, 7.74)	7.03 (6.67, 7.66)	7.51 (7.25, 7.83)
Postoperative ln(ALT)		6.53 ± 0.80	6.50 ± 0.79	6.66 ± 0.84	0.438	6.47 ± 0.87	6.40 ± 0.90	6.78 ± 0.71
Postoperative inflammatory/Nutritional indices								
Postoperative SII		1291.15 (752.37, 2363.94)	1327.66 (752.37, 2469.38)	1078.30 (752.80, 1660.59)	0.347	1277.25 (765.17, 2593.79)	1277.25 (749.87, 2474.61)	2090.23 (943.46, 3234.90)
Postoperative CLR		135.87 (65.37, 300.29)	154.56 (73.48, 314.10)	118.52 (18.07, 213.13)	0.205	142.55 (59.82, 318.52)	157.73 (56.61, 307.68)	83.80 (76.43, 282.61)
Postoperative NLR		24.96 (16.64, 37.11)	25.05 (17.45, 37.11)	23.48 (13.28, 38.94)	0.654	27.16 (17.04, 39.46)	25.61 (17.22, 38.43)	35.46 (20.99, 56.51)
Postoperative PLR		165.00 (91.98, 283.65)	171.43 (109.30, 296.15)	146.55 (73.41, 192.29)	0.122	195.43 (103.55, 292.43)	211.46 (126.19, 293.97)	133.02 (77.91, 228.97)
Postoperative SIRI		8.94 (4.58, 14.83)	8.74 (4.67, 14.83)	9.31 (3.70, 19.06)	0.813	9.24 (6.53, 26.98)	8.87 (5.92, 24.10)	14.24 (9.64, 35.84)
Postoperative PNI		41.75 (37.17, 44.42)	41.95 (38.60, 44.42)	39.60 (35.59, 44.45)	0.619	39.52 ± 4.04	39.92 ± 4.29	37.77 ± 2.24
Postoperative CAR		1.30 (0.57, 2.27)	1.30 (0.66, 2.24)	1.20 (0.27, 2.34)	0.641	1.23 (0.59, 2.24)	1.23 (0.67, 2.18)	1.43 (0.43, 3.35)
Postoperative SIRI-P		487.38 (248.52, 1050.97)	487.38 (269.22, 1024.97)	440.88 (144.75, 1198.43)	0.817	442.02 (198.94, 1168.79)	402.24 (191.11, 1070.07)	514.69 (377.27, 1818.81)

Data are presented as *n* (%) for categorical variables, mean ± SD for normally distributed continuous variables, or median (Q1, Q3) for non-normally distributed continuous variables. Distributional form was assessed separately within each cohort; therefore, the same variable may be summarized differently in the training and hold-out subsets. All postoperative laboratory variables were obtained from the first urgent postoperative blood test, generally within 1 h after completion of LT. Continuous variables were compared using Welch’s *t* test or the Wilcoxon rank-sum test, as appropriate; categorical variables were compared using the chi-square test or Fisher’s exact test. *P* values are reported for the training cohort only. *P* values were not calculated for the hold-out subset because it contained only six DVT events and group comparisons would be severely underpowered. *p* values in bold indicate *p* < 0.05. Blank *p*-value cells indicate continuation rows for categorical variables or unavailable testing because of no between-group variation. APTT, activated partial thromboplastin time; ALT, alanine aminotransferase; AST, aspartate aminotransferase; BMI, body mass index; CAR, C-reactive protein-to-albumin ratio; CLR, C-reactive protein-to-lymphocyte ratio; CRP, C-reactive protein; DVT, deep vein thrombosis; eGFR, estimated glomerular filtration rate; LT, liver transplantation; MELD, Model for End-Stage Liver Disease; NLR, neutrophil-to-lymphocyte ratio; PLR, platelet-to-lymphocyte ratio; PNI, prognostic nutritional index; PT, prothrombin time; PTA, prothrombin time activity; SD, standard deviation; SII, systemic immune-inflammation index; SIRI, systemic inflammation response index; SIRI-P, systemic inflammation response index-to-platelet ratio; ln(D-dimer), natural logarithm-transformed postoperative D-dimer; ln(AST), natural logarithm-transformed postoperative aspartate aminotransferase; ln(ALT), natural logarithm-transformed postoperative alanine aminotransferase.

In the training cohort, recipients who developed DVT differed from those without DVT with respect to MELD score, preoperative PT, postoperative ln (D-dimer), anhepatic phase, intraoperative plasma transfusion, and postoperative APTT. The hold-out subset is presented descriptively without within-subset *p* values because it contained only six DVT events and such comparisons would be severely underpowered.

### 3.2 Variable selection and logistic model derivation

**Figure 1:**
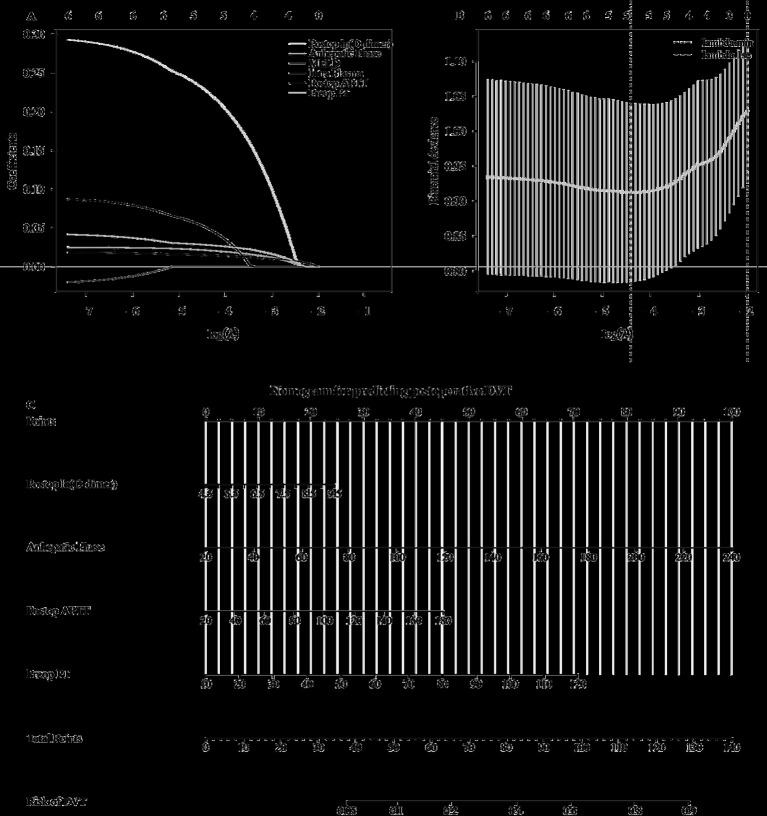
Predictor selection and nomogram construction. Panel **(A)** shows LASSO coefficient profiles for the six variables that met the exploratory training-cohort *p* < 0.05 screening criterion. Panel **(B)** shows 10-fold cross-validated binomial deviance; predictors were standardized, alpha was set to 1, and the minimum-deviance criterion (lambda.min) was used. At lambda.min, five predictors had nonzero coefficients: postoperative ln(D-dimer), anhepatic phase, intraoperative plasma transfusion, postoperative APTT, and preoperative PT. After univariable logistic regression, the four predictors with *p* < 0.10 were entered into the final multivariable model. Panel **(C)** presents the resulting logistic regression-based nomogram. APTT, activated partial thromboplastin time; DVT, deep vein thrombosis; LASSO, least absolute shrinkage and selection operator; PT, prothrombin time.

Exploratory training-cohort comparisons identified six variables with *p* < 0.05 for entry into LASSO: MELD score, preoperative PT, postoperative ln(D-dimer), anhepatic phase, intraoperative plasma transfusion, and postoperative APTT. At lambda.min, LASSO retained five predictors with nonzero coefficients, excluding MELD score: postoperative ln(D-dimer), anhepatic phase, intraoperative plasma transfusion, postoperative APTT, and preoperative PT. The coefficient profile and 10-fold cross-validation plots are shown in Figures 1A,B, respectively. These five variables were then evaluated using univariable logistic regression. Variables with *p* < 0.10 were advanced to the multivariable logistic regression model. The final logistic regression-based nomogram is shown in Figure 1C, and the corresponding univariable and multivariable regression results are presented in Tables 4, 5.

**Table 4:** Univariable logistic regression analysis of LASSO-selected candidate predictors in the training cohort.

Variable	OR	95% CI	*P* value
Postoperative ln(D-dimer)	2.123	1.212–3.720	**0.009**
Anhepatic phase	1.022	1.001–1.044	**0.043**
Intraoperative plasma transfusion	1.066	0.916–1.242	0.409
Postoperative APTT	1.020	1.007–1.032	**0.002**
Preoperative PT	1.068	0.996–1.145	0.064

Odds ratios (ORs) and 95% confidence intervals (CIs) were estimated using univariable logistic regression in the training cohort after LASSO selection at lambda.min. Predictors with *P* < 0.10 were entered into the multivariable logistic regression model. *p* values in bold indicate *p* < 0.05. APTT, activated partial thromboplastin time; CI, confidence interval; LASSO, least absolute shrinkage and selection operator; ln(D-dimer), natural logarithm-transformed postoperative D-dimer; OR, odds ratio; PT, prothrombin time.

**Table 5:** Multivariable logistic regression model for postoperative lower extremity DVT in the training cohort.

Variable	*β*	SE	OR	95% CI	*P* value
Postoperative ln(D-dimer)	0.277	0.324	1.319	0.699–2.486	0.393
Anhepatic phase	0.026	0.012	1.026	1.002–1.050	**0.032**
Postoperative APTT	0.016	0.007	1.016	1.001–1.031	**0.033**
Preoperative PT	0.036	0.024	1.037	0.988–1.088	0.138

Variables with *p* < 0.10 in the univariable analysis were entered into the final multivariable logistic regression model. Beta coefficients, standard errors (SEs), odds ratios (ORs), and 95% confidence intervals (CIs) were derived from that model in the training cohort. Individual multivariable *p* values were not used for backward elimination. *p* values in bold indicate *P* < 0.05. APTT, activated partial thromboplastin time; CI, confidence interval; ln(D-dimer), natural logarithm-transformed postoperative D-dimer; OR, odds ratio; PT, prothrombin time; SE, standard error.

In univariable logistic regression, postoperative ln (D-dimer) (OR 2.123, 95% CI 1.212–3.720; *p* = 0.009), anhepatic phase (OR 1.022, 95% CI 1.001–1.044; *p* = 0.043), postoperative APTT (OR 1.020, 95% CI 1.007–1.032; *p* = 0.002), and preoperative PT (OR 1.068, 95% CI 0.996–1.145; *p* = 0.064) met the prespecified *p* < 0.10 criterion and were entered into the multivariable model. Intraoperative plasma transfusion did not meet this criterion (OR 1.066, 95% CI 0.916–1.242; *p* = 0.409) and was not included in the final multivariable analysis.

The final multivariable logistic regression model therefore incorporated postoperative ln (D-dimer), anhepatic phase, postoperative APTT, and preoperative PT. In the adjusted model, anhepatic phase (OR 1.026, 95% CI 1.002–1.050; *p* = 0.032) and postoperative APTT (OR 1.016, 95% CI 1.001–1.031; *p* = 0.033) remained statistically significant. Postoperative ln (D-dimer) and preoperative PT were retained because the prespecified modeling sequence advanced all LASSO-selected variables with univariable *p* < 0.10; individual multivariable *p* values were not used for backward elimination.

### 3.3 Internal validation and comparative model performance

**Figure 2:**
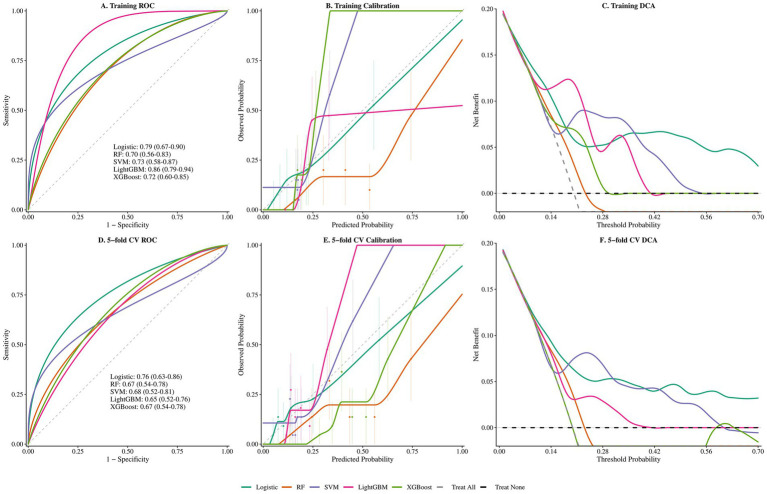
Discrimination, calibration, and decision-analytic net benefit of the five prediction models. Panels **(A–C)** show the training-cohort ROC curves, grouped calibration plots, and DCA, respectively. Panels **(D–F)** show the corresponding stratified 5-fold cross-validation results across the entire analytical cohort using out-of-fold predicted probabilities. AUCs and confidence intervals were calculated from empirical ROC objects; binormal smoothing was used only for curve display. Calibration points represent four to six equal-frequency groups according to sample size and include approximate 95% intervals for observed proportions; monotone interpolation was used only for visualization. DCA was calculated from empirical net-benefit estimates across threshold probabilities of 0.01–0.70, with locally estimated scatterplot smoothing applied only to the displayed model curves. The 95% confidence intervals for the cross-validated AUCs in Panel **(D)** were estimated using 2,000 stratified bootstrap replicates. The four-predictor set was fixed during cross-validation and predictor selection was not repeated within individual folds. The machine-learning analyses are exploratory because of the limited number of events. AUC, area under the ROC curve; CI, confidence interval; DCA, decision curve analysis; LightGBM, light gradient boosting machine; RF, random forest; ROC, receiver operating characteristic; SVM, support vector machine; XGBoost, extreme gradient boosting.

The principal discrimination and internal-validation results are summarized in Figure 2, whereas the descriptive hold-out and bootstrap analyses are presented in Supplementary Figure S2. In the training cohort, the AUCs were 0.79 (95% CI, 0.67–0.90) for logistic regression, 0.70 (0.56–0.83) for random forest, 0.73 (0.58–0.87) for support vector machine, 0.86 (0.79–0.94) for LightGBM, and 0.72 (0.60–0.85) for XGBoost (Figure 2A). In the small hold-out subset, the corresponding descriptive AUCs were 0.81 (95% CI, 0.56–1.00), 0.78 (0.59–0.96), 0.64 (0.26–1.00), 0.79 (0.61–0.96), and 0.82 (0.67–0.98), respectively (Supplementary Figure S2A); these imprecise estimates were not used for model ranking. In stratified 5-fold cross-validation across the entire cohort, based on out-of-fold probabilities, the AUCs were 0.76 (95% CI, 0.63–0.86) for logistic regression, 0.67 (0.54–0.78) for random forest, 0.68 (0.52–0.81) for support vector machine, 0.65 (0.52–0.76) for LightGBM, and 0.67 (0.54–0.78) for XGBoost (Figure 2D). In the 200-resample optimism analysis, the corresponding apparent and optimism-corrected AUCs were 0.80 and 0.76 for logistic regression, 0.90 and 0.80 for random forest, 0.77 and 0.71 for support vector machine, 0.85 and 0.75 for LightGBM, and 0.88 and 0.77 for XGBoost (Supplementary Figures S2D–F). Wide, overlapping confidence intervals and model-specific optimism did not support reliable algorithm ranking.

Threshold-dependent classification and numerical calibration results for the training cohort and descriptive pooled analytical-cohort estimates are reported in Table 6; these measures were not reported for the hold-out subset because it contained only six DVT events. The pooled estimates combine development-sample and hold-out predictions and therefore should not be interpreted as independent or fully out-of-sample validation. At the Youden probability threshold, the logistic model achieved an accuracy of 0.85, sensitivity of 0.50, specificity of 0.94, positive predictive value of 0.67, and negative predictive value of 0.88 in the training cohort. In the pooled analytical cohort, the corresponding values were 0.70, 0.81, 0.67, 0.38, and 0.93. It’s Brier score was 0.121 in the training cohort and 0.119 in the pooled dataset; the pooled calibration intercept was 0.108, the calibration slope was 1.042, and the Hosmer-Lemeshow *p* value was 0.076. In these descriptive analyses, the Hosmer-Lemeshow test indicated lack of fit for random forest and LightGBM; these findings were interpreted cautiously because the test is unstable with small event counts and should not be used to rank models. The training-cohort and 5-fold cross-validated calibration and DCA results are shown in Figures 2B,C and Figures 2E,F, respectively. The hold-out calibration and DCA plots are retained only as descriptive supplementary analyses in Supplementary Figures S2B,C, and the bootstrap distributions and optimism estimates are shown in Supplementary Figures S2D,F.

**Table 6:** Classification performance and calibration of the five prediction models in the training cohort and pooled analytical cohort.

Cohort	Model	N/DVT events	Probability threshold	Accuracy	Sensitivity (95% CI)	Specificity (95% CI)	PPV	NPV	Brier score	Calibration intercept	Calibration slope	HL *χ*^2^	HL *P*-value
Training	Logistic	99/20	0.387	0.848	0.500 (0.299–0.701)	0.937 (0.860–0.973)	0.667	0.881	0.1212	0.0000	1.0000	9.933	0.270
Training	RF	99/20	0.672	0.808	0.450 (0.258–0.658)	0.899 (0.813–0.948)	0.529	0.866	0.2045	−1.2700	0.8842	36.167	**<0.001**
Training	SVM	99/20	0.249	0.869	0.500 (0.299–0.701)	0.962 (0.894–0.987)	0.769	0.884	0.1328	2.5129	2.9119	12.758	0.120
Training	LightGBM	99/20	0.213	0.778	0.900 (0.699–0.972)	0.747 (0.641–0.830)	0.474	0.967	0.1349	2.7151	3.3105	22.470	**0.004**
Training	XGBoost	99/20	0.222	0.707	0.650 (0.433–0.819)	0.722 (0.614–0.808)	0.371	0.891	0.1524	3.0799	3.3262	11.560	0.172
Pooled cohort	Logistic	131/26	0.125	0.695	0.808 (0.621–0.915)	0.667 (0.572–0.750)	0.375	0.933	0.1186	0.1081	1.0420	14.239	0.076
Pooled cohort	RF	131/26	0.509	0.817	0.885 (0.710–0.960)	0.800 (0.714–0.865)	0.523	0.966	0.1524	−1.5242	2.0647	36.865	**<0.001**
Pooled cohort	SVM	131/26	0.183	0.824	0.577 (0.389–0.745)	0.886 (0.811–0.933)	0.556	0.894	0.1317	2.5459	2.9366	15.139	0.056
Pooled cohort	LightGBM	131/26	0.213	0.771	0.808 (0.621–0.915)	0.762 (0.672–0.833)	0.457	0.941	0.1355	2.3233	2.8863	31.165	**<0.001**
Pooled cohort	XGBoost	131/26	0.206	0.710	0.692 (0.500–0.835)	0.714 (0.622–0.792)	0.375	0.904	0.1499	3.4989	3.6141	9.565	0.297

Continuous predictor variables were retained on their continuous scales (except for the prespecified natural-log transformation of D-dimer) and were not dichotomized using data-driven cut-offs. The values in the Probability threshold column are model-classification thresholds selected by the Youden index and are not predictor cut-offs. Separate thresholds were estimated for the training cohort and pooled analytical cohort. Accuracy, sensitivity, specificity, PPV, and NPV were calculated at the corresponding threshold; values in parentheses for sensitivity and specificity are Wilson 95% confidence intervals. Random-forest training probabilities were based on out-of-bag votes, whereas the other training-cohort values are apparent estimates. The pooled analytical-cohort results combine development-sample and hold-out predictions from models fitted in the training cohort; because they include development observations, they are descriptive and should not be interpreted as independent or fully out-of-sample validation. Threshold-dependent classification and numerical calibration measures were not reported for the hold-out subset alone because it contained only six DVT events. Brier score was calculated as the mean squared difference between the observed outcome and predicted probability. Calibration intercept and slope were estimated by logistic recalibration of the outcome on the logit of the predicted probability. The Hosmer-Lemeshow test used 10 equal-frequency groups, with automatic reduction when required by sample size. Bold HL *P* values indicate evidence of lack of fit at *P* < 0.05. For the training-cohort logistic model, a calibration intercept of 0 and slope of 1 are expected because recalibration was assessed in the same data used to fit the model; these apparent values should not be interpreted as evidence of external calibration. DVT, deep vein thrombosis; HL, Hosmer–Lemeshow; LightGBM, light gradient boosting machine; NPV, negative predictive value; PPV, positive predictive value; RF, random forest; ROC, receiver operating characteristic; SVM, support vector machine; XGBoost, extreme gradient boosting; *χ*^2^, chi-square.

### 3.4 Exploratory model interpretation

**Figure 3:**
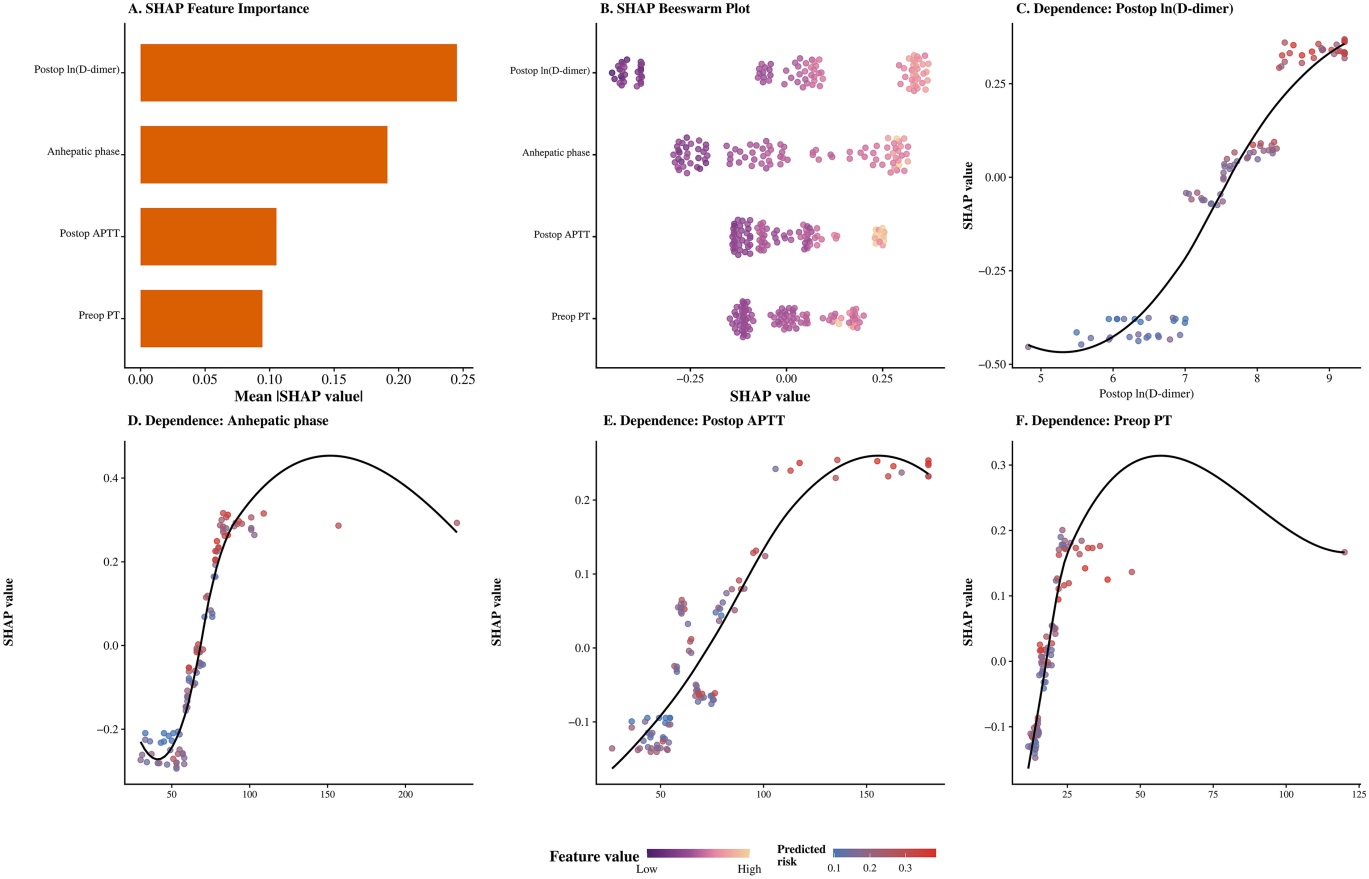
Global SHAP importance and dependence analysis for the exploratory LightGBM model. SHAP contribution values were calculated from the originally developed training-cohort LightGBM model on the raw-score scale and aggregated from encoded features to their parent variables. Panel **(A)** shows mean absolute SHAP feature importance. Panel **(B)** shows the beeswarm distribution of individual contributions, with color representing the scaled observed feature value. Panels **(C–F)** present dependence plots with locally estimated scatterplot smoothing for postoperative ln(D-dimer), anhepatic phase, postoperative APTT, and preoperative PT, respectively; point color represents the LightGBM-predicted risk. SHAP was used only for *post hoc* interpretation and did not contribute to predictor selection or nomogram construction. These analyses describe model behavior and should not be interpreted as causal effects. APTT, activated partial thromboplastin time; LightGBM, light gradient boosting machine; PT, prothrombin time; SHAP, Shapley additive explanations.

The principal exploratory model-interpretation analyses are summarized in Figure 3, with additional SHAP visualizations in Supplementary Figure S3. SHAP contribution values were calculated in the training cohort from the originally developed LightGBM model and aggregated from encoded features to the four parent predictors. Global importance was defined by the mean absolute SHAP value (Figure 3A). In the beeswarm plot, point color denotes the scaled observed feature value (Figure 3B). The four enlarged dependence plots use locally estimated scatterplot smoothing to display model behavior for postoperative ln(D-dimer), anhepatic phase, postoperative APTT, and preoperative PT; point color denotes the LightGBM-predicted risk (Figures 3C,F). The supplementary heatmap orders recipients from higher to lower predicted risk (Supplementary Figures S3A). The representative waterfall plots show the DVT case with the highest predicted risk and the non-DVT recipient with the lowest predicted risk within the training cohort (Supplementary Figures S3B,C). These analyses were *post hoc* and did not contribute to LASSO selection, logistic-regression fitting, or nomogram construction.

The dependence plots suggested that the LightGBM model output generally increased with higher postoperative ln (D-dimer) and postoperative APTT, whereas anhepatic phase and preoperative PT showed nonlinear patterns. These analyses describe the behavior of one exploratory algorithm and do not establish causal effects or demonstrate superiority over the logistic model.

## 4 Discussion

In this retrospective cohort of adult LT recipients, we developed an immediate postoperative logistic regression-based nomogram for lower-extremity DVT. Exploratory training-cohort comparisons reduced the candidate set to six variables; standardized 10-fold LASSO at lambda.min retained five nonzero candidates, and the four variables meeting the prespecified univariable logistic-regression criterion of *p* < 0.10 entered the final multivariable model. Internal assessment included a descriptive hold-out subset, stratified 5-fold cross-validation based on out-of-fold probabilities, and 200-resample bootstrap optimism correction using the fixed four-predictor set. The 5-fold cross-validated and optimism-corrected AUCs for logistic regression were both approximately 0.76. Although some flexible algorithms achieved higher apparent AUCs in selected analyses, their optimism and calibration instability were greater, and the wide, overlapping confidence intervals did not support reliable model ranking. The machine-learning analyses, including the SHAP analysis of the originally developed LightGBM model, should therefore be considered exploratory. The logistic model was used for nomogram construction because it provides a transparent and clinically interpretable risk equation, not because it was statistically superior.

The observed DVT rate of 19.8% should be interpreted in light of the postoperative ultrasound assessment strategy. All included recipients underwent at least one bilateral duplex examination within the first 7 postoperative days, although examinations were not scheduled daily and the timing and number of repeat examinations varied according to routine care and clinical condition. This approach was broader than purely symptom-triggered testing and may have increased detection of clinically silent or distal thromboses. However, the non-uniform examination schedule may also have influenced event ascertainment and complicates direct comparison with cohorts using a fixed screening time point or imaging only when clinically indicated.

The association between a longer anhepatic phase and DVT is clinically relevant because it is specific to transplantation and may summarize operative complexity, venous stasis, ischemic burden, hemodynamic instability, and endothelial injury (6, 22). Its inclusion distinguishes the present model from generic surgical VTE scores. Nevertheless, the association should not be interpreted causally, and its coefficient may vary with surgical technique, center experience, graft characteristics, and perioperative management.

Perioperative antithrombotic management after LT is further complicated by pre-existing portal vein thrombosis, vascular reconstruction, and competing bleeding risk. Prior studies have emphasized the importance of portal vein thrombosis grade, screening, and reconstruction-related factors, while not supporting indiscriminate therapeutic anticoagulation in all recipients (23–25).

Coagulation-related variables after LT remain biologically plausible but require cautious interpretation. Advanced liver disease is characterized by rebalanced hemostasis rather than uniform hypocoagulability (1–4). Surgical trauma, reperfusion injury, transfusion, endothelial activation, and early graft dysfunction may further destabilize this balance (6, 12). Conventional PT and APTT provide only a limited representation of global hemostatic balance in advanced liver disease (26–28). D-dimer may integrate thrombin generation, fibrinolysis, inflammation, and tissue injury (29–34). In the present study, the analyzed postoperative sample was the first urgent blood test, generally obtained within 1 h after LT and before initiation of postoperative pharmacologic anticoagulation according to routine workflow. This timing reduces concern about postoperative anticoagulant exposure; however, intraoperative anticoagulants, transfusion, factor consumption, and subclinical thrombosis already present at sampling may still have influenced APTT and D-dimer.

From a clinical perspective, the nomogram should be viewed as a surveillance and reassessment aid rather than an automatic trigger for anticoagulation. Current evidence and expert guidance favor individualized rather than indiscriminate anticoagulation or thromboprophylaxis in complex hepatic populations (12–14, 35). A practical pathway would combine the estimated risk with Doppler findings, bleeding status, platelet count, graft and renal function, vascular reconstruction, mobility, and pre-existing thrombotic indications. Potential implementation barriers include the need for external calibration, consistent timing and assay methods for the first postoperative blood sample, integration into electronic clinical workflows, access to timely postoperative ultrasound assessment, operator dependence, and uncertainty regarding the intervention threshold at which net benefit would outweigh bleeding risk.

Our findings complement emerging LT-specific prediction research. An et al. developed a nomogram for a broader composite of postoperative thrombosis and identified intraoperative blood loss as an important predictor (36). A prospective anticoagulation stratification algorithm has also been evaluated after LT, underscoring the need to balance bleeding and thrombotic risks rather than rely on a prediction score alone (37). Li et al. developed a VTE nomogram incorporating clinical and postoperative laboratory variables, including the D-dimer/fibrinogen ratio (38). Xiao et al. constructed a lower-extremity DVT model based on smoking history, hyperlipidemia, intraoperative blood loss, and D-dimer (39). A recent single-center study identified age, diabetes, ascites, hepatic encephalopathy, and postoperative D-dimer as predictors (40). In contrast, the present model focuses on measurements available immediately after surgery, generally within 1 h, and incorporates the LT-specific anhepatic phase. Differences in case mix, ultrasound screening intensity, outcome windows, prophylaxis practices, predictor timing, and validation strategies limit direct performance comparisons across studies.

This study has several important limitations. First, only 26 DVT events occurred overall and 20 occurred in the training cohort; with four variables in the final multivariable model, the events-per-variable ratio was approximately five. The limited effective sample size increases coefficient uncertainty and the risk of overfitting, particularly for flexible machine-learning algorithms. The staged sequence of exploratory group comparison, LASSO, univariable logistic screening, and conventional multivariable logistic estimation remains susceptible to sample-specific selection and coefficient instability. A penalized final estimator was considered but was not implemented; LASSO feature selection alone does not eliminate overfitting in the final conventional logistic model. Moreover, the selected predictor set was held fixed rather than reselected within each cross-validation fold or bootstrap resample. Consequently, the reported validation estimates assess the fixed model specification and may underestimate optimism in the complete selection pipeline. The use of 200 bootstrap resamples also limits the precision of the optimism estimate, and neither cross-validation nor bootstrap correction can substitute for a larger development sample. Second, the study was retrospective and single-center. Our center established its LT program relatively recently, and no harmonized external cohort was available during the revision period. The hold-out subset was derived from the same source cohort and does not constitute independent external validation. Third, individualized thromboprophylaxis may have introduced residual treatment confounding. Although the first urgent postoperative blood sample was generally obtained before postoperative pharmacologic anticoagulation, exact intraoperative and very early postoperative anticoagulant exposure could not be reconstructed for every recipient. Fourth, postoperative ultrasonography was not performed according to a uniform daily schedule; variation in examination timing and frequency may have influenced event ascertainment, and exclusion of patients without the required postoperative ultrasound may have introduced selection bias. Fifth, advanced viscoelastic measurements such as thromboelastography (TEG) and rotational thromboelastometry (ROTEM), as well as serial postoperative biomarker trajectories, were unavailable and could not be incorporated. Finally, the training and pooled analytical-cohort classification and calibration measures in Table 6 are descriptive and partly apparent; calibration estimates remain imprecise because of the small event count, and SHAP analyses describe model behavior rather than causal mechanisms.

Future research should prioritize prospective multicenter recruitment with prespecified systematic outcome assessment, adequate event numbers, and external validation across centers with different surgical and prophylaxis practices. Model updating should evaluate serial postoperative measurements, TEG or ROTEM variables, and clinically meaningful DVT phenotypes. Comparative studies should also determine whether risk-guided ultrasound surveillance or prophylaxis improves patient-important outcomes without increasing bleeding. Until such evidence is available, the present nomogram should be regarded as a preliminary internally assessed tool rather than a ready-to-deploy clinical decision rule.

## 5 Conclusion

We developed an immediate postoperative nomogram incorporating postoperative ln (D-dimer), anhepatic phase, postoperative APTT, and preoperative PT for estimating lower-extremity DVT risk within 7 days after LT. Internal assessment suggested moderate discrimination, but the limited event count, non-nested predictor selection, and wide, overlapping model confidence intervals preclude claims of algorithmic superiority. The nomogram may support risk-stratified surveillance and multidisciplinary reassessment; prospective multicenter external validation is required before routine clinical use.

## Data Availability

The raw data supporting the conclusions of this article will be made available by the authors, without undue reservation.

## Supplementary material

SUPPLEMENTARY FIGURE 1 Study flow chart of patient selection and cohort allocation. A total of 145 adult recipients who underwent LT between August 2023 and January 2026 were screened. Fourteen recipients were excluded because of preexisting lower-extremity thrombosis (*n* = 1), missing preoperative ultrasound results (*n* = 4), or missing postoperative ultrasound results (*n* = 9). The final analytical cohort included131recipients, who were divided by outcome-stratified random sampling into a training cohort (*n* = 99)and a hold-out subset (*n* = 32) at a ratio of 3:1.The outcome was newly detected lower-extremity DVT identified by bilateral duplex color Doppler ultrasonography within the first 7 days after LT. DVT, deep vein thrombosis; LT, liver transplantation.

SUPPLEMENTARY FIGURE 2 Descriptivehold-outanalysesandbootstrapoptimismcorrectionforthefiveprediction models. Panels **(A–C)** show the ROC curves, grouped calibration plots, and DCA in the hold-out subset, respectively. Because this subset contained only six DVT events, these panels are provided for descriptive completeness and were not used for reliable model ranking or numerical calibration inference. Panel **(D)** shows the distributions of bootstrap-sample apparent AUCs from 200 resamples. Panel **(E)** compares the full-data apparent and optimism-corrected AUCs, and Panel **(F)** shows the estimated mean optimism. Predictor selection was not repeated within bootstrap samples, and the four-predictor set was fixed. The displayed ROC, calibration, and DCA curves use the same graphical smoothing procedures described for Figure 2. The machine-learning analyses are exploratory because of the limited number of events. AUC, area under the ROC curve; DCA, decision curve analysis; LightGBM, light gradient boosting machine; RF, random forest; ROC, receiver operating characteristic; SVM, support vector machine; XGBoost, extreme gradient boosting.

SUPPLEMENTARY FIGURE 3 Additional SHAP visualizations for the exploratory LightGBM model. Panel **(A)** presents the SHAP heatmap, with recipients ordered from higher to lower LightGBM-predicted risk. Panel **(B)** shows the waterfall explanation for the DVT case with the highest predicted risk in the training cohort, and Panel **(C)** shows the corresponding explanation for the non-DVT recipient with the lowest predicted risk. Positive SHAP contributions increase the model raw score, whereas negative contributions decrease it. These plots provide cohort-level and individual descriptions of the originally developed training-cohort LightGBM model and should not be interpreted as causal effects. DVT, deep vein thrombosis; LightGBM, light gradient boosting machine; SHAP, Shapley additive explanations.

## Glossary

ALT: Alanine aminotransferase
APTT: Activated partial thromboplastin time
AST: Aspartate aminotransferase
AUC: Area under the receiver operating characteristic curve
BMI: Body mass index
CAR: C-reactive protein-to-albumin ratio
CI: Confidence interval
CLR: C-reactive protein-to-lymphocyte ratio
CRP: C-reactive protein
DCA: Decision curve analysis
DVT: Deep vein thrombosis
eGFR: Estimated glomerular filtration rate
LASSO: Least absolute shrinkage and selection operator
LightGBM: Light gradient boosting machine
ln: Natural logarithm
LT: Liver transplantation
ln(D-dimer): Natural logarithm-transformed D-dimer
ln(AST): Natural logarithm-transformed aspartate aminotransferase
ln(ALT): Natural logarithm-transformed alanine aminotransferase
MELD: Model for End-Stage Liver Disease
NLR: Neutrophil-to-lymphocyte ratio
NPV: Negative predictive value
OR: Odds ratio
PLR: Platelet-to-lymphocyte ratio
PNI: Prognostic nutritional index
PPV: Positive predictive value
PT: Prothrombin time
PTA: Prothrombin time activity
Q1: First quartile
Q3: Third quartile
RF: random forest
ROC: Receiver operating characteristic
ROTEM: Rotational thromboelastometry
SD: Standard deviation
SE: Standard error
SHAP: Shapley additive explanations
SII: Systemic immune-inflammation index
SIRI: Systemic inflammation response index
SIRI-P: Systemic inflammation response index-to-platelet ratio
SVM: Support vector machine
TEG: Thromboelastography
TRIPOD: Transparent Reporting of a Multivariable Prediction Model for Individual Prognosis or Diagnosis
VIF: Variance inflation factor
VTE: Venous thromboembolism
XGBoost: Extreme gradient boosting.
